# A Multicentric Study on Adverse COVID-19 Outcomes Among Pregnant and Nonpregnant Women in Multidisciplinary Hospitals of Kazakhstan

**DOI:** 10.3390/diagnostics15070900

**Published:** 2025-04-01

**Authors:** Zhansaya Nurgaliyeva, Lyudmila Pivina, Sharapat Moiynbayeva, Galiya Alibayeva, Meruyert Suleimenova, Nailya Kozhekenova, Moldir Abdullina, Maulen Malgazhdarov, Mira Turbekova, Dejan Nikolic, Milan Lackovic, Antonio Sarria-Santamera, Milena Santric-Milicevic

**Affiliations:** 1 Faculty of Medicine and Health Care, Al-Farabi Kazakh National University, Almaty 050044, Kazakhstan; nailyakozhekenova@gmail.com (N.K.); 11monti@mail.ru (M.A.); 2 Department of Internal Medicine, Semey Medical University, Semey 071407, Kazakhstan; lyudmila.pivina@smu.edu.kz; 3 Department of Science and Consulting, Kazakhstan Medical University «KSPH», Almaty 050044, Kazakhstan; moyinbaeva@inbox.ru; 4 Semey Emergency Hospital, Semey 071407, Kazakhstan; galiya.5@mail.ru; 5 Department of Science, S.D. Asfendiyarov Kazakh National Medical University, Almaty 050044, Kazakhstan; meruyert.sd@gmail.com; 6 Department of Surgical Diseases No. 2, Kazakh-Russian Medical University, Almaty 050044, Kazakhstan; maulen-ms@mail.ru; 7 Karasai Interdistrict Multidisciplinary Hospital, Almaty 050044, Kazakhstan; 8 Department of Clinical Disciplines, Al-Farabi Kazakh National University, Almaty 050044, Kazakhstan; meerakz@mail.ru; 9 Faculty of Medicine, University of Belgrade, 11000 Belgrade, Serbia; dejan.nikolic@udk.bg.ac.rs; 10 Department of Physical Medicine and Rehabilitation, University Children’s Hospital, 11000 Belgrade, Serbia; 11 University Hospital “Dragisa Misovic”, 11000 Belgrade, Serbia; milan.lackovic@dragisamisovic.bg.ac.rs; 12 School of Medicine, Nazarbayev University, Astana 010000, Kazakhstan; antonio.sarria@nu.edu.kz; 13 School of Public Health, Nazarbayev University, Astana 010000, Kazakhstan; 14 Laboratory for Strengthening the Health System and Workforce for Health Equity, Institute of Social Medicine, Faculty of Medicine, University of Belgrade, 11000 Belgrade, Serbia

**Keywords:** pregnancy, COVID-19, severity, adverse outcomes, propensity score matching, inpatient care

## Abstract

**Background and Objectives:** The study aimed at identification and analysis of adverse COVID-19 outcomes (admission to intensive care units due to COVID-19, acute respiratory distress syndrome, mechanical ventilation, and death) among hospitalized pregnant and nonpregnant women, which are critical for informed decision-making in obstetric diagnostics and healthcare. **Materials and Methods:** This was a retrospective observational study conducted on a series of inpatient pregnant women comparatively followed up with nonpregnant women hospitalized between 15 July 2020 to 20 January 2022 across multidisciplinary hospitals in three cities of Kazakhstan. Following group matching with propensity score for COVID-19 disease severity, residence status, and age, the study ultimately included 156 participants, of whom 50% were pregnant, from an initial sample of 314 female inpatients diagnosed with COVID-19. All findings were considered statistically significant at a *p*-value < 0.05. **Results:** Laboratory investigations revealed significantly elevated levels of erythrocyte sedimentation rate, creatinine, neutrophils, platelet count, alanine aminotransferase, aspartate aminotransferase, lymphocyte count, and C-reactive protein in pregnant inpatients compared to nonpregnant inpatients. Furthermore, pregnant women exhibited significantly higher levels of D-dimer (2402.97 ng/mL vs. 793.91 ng/mL) and procalcitonin (0.398 ng/mL vs. 0.134 ng/mL) compared to their nonpregnant counterparts. Overall, 16.88% of the pregnant women were admitted to the intensive care unit, whereas among the nonpregnant women, only 2.6% were hospitalized. The most lethal outcomes (8.3%) occurred among pregnant women, while for nonpregnant women, there were two cases (1.3%). **Conclusions:** Pregnant women diagnosed with COVID-19 may exhibit more severe clinical symptoms and encounter more adverse outcomes compared to their nonpregnant counterparts. Future research should incorporate larger matched samples to comprehensively explore the association between additional factors and clinical conditions.

## 1. Introduction

As the world transitions beyond the coronavirus disease (COVID-19) pandemic, a United Nations Sustainable Development Goals report underscores the critical importance of early detection and treatment of pregnancy disorders to promote healthy outcomes for both mothers and their children after the COVID-19 pandemic [1]. This is because the global maternal mortality ratio exceeds the goal for 2030 by more than threefold, and it is projected that 35 million children will not reach their fifth birthday without swift intervention by then [1]. COVID-19 continuously evolves, and new variants are anticipated to emerge over time [2]. Healthcare stakeholders and professionals remain concerned about the evidence suggesting that pregnant women with COVID-19 symptoms are at greater risk of preterm birth or stillbirth than those who are asymptomatic [3,4]. Additionally, those with severe symptoms may face increased chances of complications like preeclampsia and gestational diabetes, as well as preterm birth or low birth weight, compared to those with milder symptoms [5]. Moreover, it is becoming increasingly evident that COVID-19 affects the respiratory system and significantly impacts various physiological systems, including the nervous, immune, and reproductive systems [6,7]. Low- and middle-income countries face challenges in achieving optimal pregnancy outcomes for COVID-19-affected patients due to inconsistent healthcare service quality in early diagnostics, obstetric care, and delivery practices, compounded by existing socio-economic disparities [4,8,9]. An initiative established by the World Health Organization in collaboration with global partners founded upon the theory of change aimed to reduce maternal and newborn mortality rates, as well as stillbirth rates, by fifty percent by 2022. This initiative underscored the necessity for national obstetric programs to incorporate routine patient and process outcome indicator monitoring [9]. In part due to a lack of valid data, negative COVID-19 outcomes among pregnant and nonpregnant women remain inadequately explored in low- and middle-income countries [3,10]. Given the multiple impact of COVID-19 [6,7], a clear understanding of negative COVID-19 outcomes in pregnant and nonpregnant women, such as the possibility of admission to an intensive care unit (ICU) [11,12], the incidence of acute respiratory distress syndrome (ARDS) [13], the necessity of mechanical ventilation [11,12], and the case fatality rate [12,14], is essential for providing healthcare recommendations within specific contexts.

This study aimed to elucidate the socio-demographic and clinical factors associated with adverse COVID-19 outcomes, including ICU admission due to COVID-19, mechanical ventilation, ARDS, and death, among pregnant and nonpregnant inpatients. At the international level, study findings can contribute evidence to burden-of-proof studies [15], while at a national level, they can inform stakeholders regarding the quality of maternal and newborn medical care and highlight the critical role that appropriately resourced early-detection measures may play in programs and protocols during crises.

### The Healthcare of Pregnant Women with COVID-19 in the Republic of Kazakhstan

In the Republic of Kazakhstan, a middle-income country, the neonatal mortality rate per 1000 births increased from 4.71 in 2019 to 5.03 in 2021, while maternal mortality was 36.5 in 2020 and 44.7 in 2021 [16]. These data indicate a pressing need for enhanced maternity care during times of crisis, as well as the critical importance of promptly differentiating between severe and nonfatal symptoms of COVID-19 in pregnant individuals. The physiological and immunological changes associated with pregnancy may predispose mothers to acute respiratory syndrome, thereby heightening the risk of adverse outcomes for both the mother and the fetus [5,17]. The Ministry of Health of Kazakhstan has established a vision for 2030 focusing on pregnant women infected with COVID-19 [16]. The report highlights that common nonfatal symptoms include fever, cough, shortness of breath, malaise, and diarrhea, occurring in 68%, 34%, 12%, 12%, and 6% of nonfatal cases, respectively [10]. Findings related to elevated body temperatures remain inconclusive [15,18], as does the understanding of signals indicating potential adverse pregnancy outcomes.

Although emergency and intensive care units were reorganized, most medical care for pregnant women with COVID-19 was provided through primary healthcare services [19,20,21,22]. This involved converting a multidisciplinary urban hospital into a designated “infectious” facility for pregnant women, women in labor, and maternity patients. The process required mobilizing all available resources and personnel, including procuring additional diagnostics, medical equipment, and medications beyond the hospital’s standard offerings while strictly adhering to the diagnosis and treatment protocols [23]. The first protocol for diagnosing and treating coronavirus infection for all categories of patients in the Republic of Kazakhstan was approved on 3 March 2020 (protocol 85). Upon its approval on 23 July 2021, the specific protocol for diagnostic and treatment methods of pregnant women, women in labor, and postpartum women was updated on 5 August 2021 and 4 March 2022 [23].

This study presents an overview of the specific protocol implemented in Kazakhstan hospitals for the diagnosis and treatment of COVID-19 in pregnant women, women in labor, and postpartum women, as well as nonpregnant women who were affected by the virus. Grounded in contemporary empirical evidence and theoretical frameworks, the findings of this study not only reflect the outcomes of the protocol but also indicate the potential for further enhancements in light of these results.

## 2. Materials and Methods

### 2.1. Study Design

This was a retrospective observational study in the Republic of Kazakhstan that explored adverse COVID-19 events among a series of inpatient pregnant women comparatively followed up with nonpregnant women, also hospitalized because of COVID-19. The study participants were all pregnant and nonpregnant women with COVID-19 who were admitted to multidisciplinary city hospitals involved in a dissertation research project at Al-Farabi Kazakh National University in Almaty, Astana, and Semey during the study period. Official requests were sent to multidisciplinary hospitals in major cities across the Republic of Kazakhstan, where specialized departments for pregnant women with confirmed coronavirus infections were established. Three multidisciplinary hospitals responded positively and agreed to provide the requested data out of the eight requests sent.

The study was conducted upon obtaining approval from the Ethics Committee of Al-Farabi Kazakh National University (approval IRB-A470, 9 June 2022). In addition, individual informed consent was not obtained due to the retrospective collection of the anonymized patient data.

### 2.2. Study Participants and COVID-19 Diagnosis

The study participants were all women of reproductive age (15–49 years old) hospitalized due to COVID-19 in the three multidisciplinary city hospitals of Almaty, Astana, and Semey from 15 July 2020 to 20 January 2022. The study time frame was meticulously selected to optimize the likelihood of capturing only a single hospitalization for COVID-19 patients while minimizing the risk of readmissions for the same condition. This approach was informed by the estimate that approximately 15% of COVID-19 inpatients test positive again following discharge [24].

In Kazakhstan, hospital physicians establish a diagnosis of COVID-19 (U07.1, U07.2) based on the results of a polymerase chain reaction (PCR) test, along with findings from a radiography (X-ray) examination or computerized tomography (CT) scan, in accordance with the national treatment protocol [23]. The severity of COVID-19 cases was assessed clinically following the criteria outlined in the national protocols [23].

Accordingly, mild COVID-19 was defined as the presence of symptoms such as normal, subfebrile, or febrile body temperature, no difficulty breathing, a respiratory rate of <20 breaths per minute, percentage saturation of hemoglobin with oxygen (SpO2) at rest >95%, no changes on X-ray or CT of the lungs, and heart rate 60–80 beats/min. The number of these cases in the study was nine among pregnant patients and one in the group of nonpregnant participants.

Moderate COVID-19 was defined as a case with an increase in body temperature (more often febrile), shortness of breath during exercise, a respiratory rate of 20–22 breaths per minute, SpO2 at rest 94–95%, X-ray foggy darkening, often of rounded morphology, with peripheral and lower distribution over the lungs, CT 1–2 lung lesions up to 50%, and heart rate 80–100 beats/min. The number of these patients in the study was 108 among pregnant patients and 147 among nonpregnant participants.

Severe COVID-19 was defined as the presence of an increase in body temperature (subfebrile, febrile, less often usual), shortness of breath with slight exertion, talking, at rest, a respiratory rate of 23–30 breaths per minute, SpO2 at rest 90–93%, X-ray signs of bilateral viral lung damage, CT 3–4 lung lesion volume >50%, and heart rate 100–120 beats/min. The number of these cases in the study was 29 among pregnant patients, and 8 among nonpregnant participants.

Critical COVID-19 was defined as an increased body temperature (subfebrile, febrile, less often usual), shortness of breath at rest, BPM > 30 per minute, SpO2 at rest < 90%, X-ray signs of significant bilateral viral lung damage, CT 4 lung lesion volume 75%–100%, heart rate more than 120 beats/min, respiratory failure requiring intubation or mechanical ventilation, septic shock, and multiple organ dysfunction or failure. The number of these cases in the study was 1. For analytical purposes, we grouped 6 persons: the first group combined mild- and moderate-severity cases, while the second group consisted of severe and critical conditions.

### 2.3. Patient Inclusion and Exclusion Criteria in the Study

The following inclusion and exclusion criteria were established to minimize potential biases in the study. The inclusion criteria in the study were:

(a) Female sex.
(b) Age between 15 and 49 years.
(c) First emergency hospitalization due to a diagnosis of COVID-19 (International Classification for Diseases, X revision: U07.1 and U07.2) in the study period.
(d) Hospitalization period 15 July 2020 to 20 January 2022.
(e) City hospitals providing emergency hospitalization for COVID-19 cases.
(f) Patients with indications for emergency hospitalization due to COVID-19, including the following based on the clinical protocol of COVID-19 diagnosis and treatment [23]:
  I. Moderate-severity COVID-19.
  II. Severe COVID-19.
  III. Extremely severe/critical (acute respiratory distress syndrome (ARDS), sepsis, septic shock, etc.), and fever (38 degrees Celsius and higher) resistant to antipyretic drugs for 3 days, or respiratory rate > 24 per 1 min/dyspnea at regular physical activity or speech/SpO2 reduction < 95% [6], or CT 3–4.

The exclusion criteria in the study were:

(a) Patients hospitalized before 15 July 2020.
(b) Inpatients in the postpartum period up to six months after labor.
(c) Inpatients who were hospitalized for reasons other than COVID-19.
(d) Inpatients transferred to other hospitals for treatments other than COVID-19 during the study.
(e) Patients who visited hospitals for a consultation and were not hospitalized.
(f) Patients whose necessary data for propensity score matching were incomplete/missing (severity of COVID-19, residence status, and age).

The sample size was determined to identify differences in proportions of negative events between nonpregnant and pregnant women with COVID-19. The literature indicates that 11.7% of pregnant women require mechanical ventilation (BiPAP) compared to 25.4% of nonpregnant women [11]. To detect these proportions with a statistical power of 0.8 and a significance level of 0.05 using the chi-squared test, a sample size of 125 participants per group is required. The mortality rate is reported to be 5.0% in pregnant women and 15.5% in nonpregnant women [11]. A sample size of 130 respondents per group is required to achieve an equivalent statistical power and significance level for this outcome. Therefore, the final recommended sample size was established by choosing the greater of the two computed values, yielding a total of 260 respondents.

According to the inclusion and exclusion criteria, 157 pregnant women requiring emergency admission for COVID-19 were identified in the group of pregnant inpatients with COVID-19. The group of nonpregnant women of reproductive age who were hospitalized due to COVID-19 comprised 157 inpatients. The number of cases that meet the eligibility criteria established by the sampling process at each city hospital reflects the availability of data, the readiness of local medical teams, and their ability to contribute to the study effectively due to the dynamics of establishing red zones in the hospitals during COVID-19 pandemic. After matching for disease severity, residence, and age, the final matched sample consisted of 78 pregnant and 78 nonpregnant women. The results of the total sample and matched results groups are presented.

The response rate for the total sample was between 87.9% and 100% and for the matched sample between 76.9% and 100%.

### 2.4. Study Variables

In this study, the selection of variables was determined by the insights derived from meta-analyses, systematic literature reviews, pertinent cohort studies, reviews, and clinical and cross-sectional studies [11,12,13,21,24,25,26,27,28,29,30,31,32,33,34,35,36]. The primary study outcomes of interest for female patients with COVID-19 were four types of COVID-19 adverse events: admission to an intensive care unit (ICU) due to COVID-19, long hospital length of stay, ARDS, and death. Additionally, for the second study objective, we conducted a separate analysis in a group of pregnant inpatients to examine the relationship between the severity of COVID-19 and the instances of stillbirth in children and subsequent admissions to the neonatal department.

In the statistical analysis, we focused on three sets of data: socio-demographic variables, pregnancy/labor characteristics upon the first urgent admission to the hospital due to COVID-19, and clinical variables. Clinical variables were composed of two subsets of information: diagnostic data taken upon admission to the hospital and treatment data for COVID-19 taken from discharge records.

Socio-demographic variables in the study were age years, age group (less than 40 years or more than 40 years), and residence (urban/rural). The pregnancy/labor characteristics that were analyzed were the following: (a) pregnancy (first, second, third trimester, labor), (b) preeclampsia (yes/no), (c) mode of delivery (vaginal delivery/cesarean), (d) labor with premature birth (yes/no), and (e) postpartum hemorrhage (yes/no).

The clinical data with regard to the diagnostic process comprised the following medical data (expected range of units in parentheses) upon patient admission to the hospital:

(a) Blood group (I–IV).
(b) CT process (one side or two sides).
(c) Stage of computerized tomography (CT 1–4). CT 1 is lung damage up to 25%, CT 2 is lung damage of 25%–50%, CT 3 is lung damage of 50%–75%, CT 4 is lung damage > 75%.
(d) Presence (yes/no) of comorbidities (arterial hypertension, diabetes, hyperglycemia, anemia).
(e) Presence of symptoms (yes/no) at admission to hospitals (fever, cough, dyspnea, myalgia, anosmia, diarrhea, sweating, weakness, chest discomfort, sore throat, hypotonia, arrhythmia, respiratory rate, heart rate, temperature).
(f) Hemoglobin level (100–140 g/l).
(g) Erythrocyte sedimentation rate (40 mm/h).
(h) Leukocytes (from 4 to 15 × 109/l, in pregnant women up to 11 × 109/l).
(i) Segmented neutrophils (45%–72%).
(j) Band neutrophils (1%–5%).
(k) Lymphocytes (19%–37%).
(l) Platelets (169–358 × 109/l).
(m) Alanine aminotransferase (alt) (up to 32.2).
(n) Aspartate aminotransferase (ast) (up to 31).
(o) Procalcitonin (up to 1 ng/ml).
(p) C-reactive protein (up to 20 mg/L).
(q) D-dimer (up to 457 ng/L).
(r) Creatinine (35–70 mmol/L).
(s) Glucose (3.3–5.1).
(t) Ferritin (up to 150 ng/mL).

For the second objective of the study, the treatment of female patients with COVID-19 was analyzed using clinical data from discharge reports, including:

(a) Treatment interventions (transfusion of blood and blood substitutes).
(b) Ventilation (invasive, non-invasive).
(c) Nasal cannula (yes/no).
(d) Extracorporeal membrane oxygenation (ECMO) (yes/no).
(e) Antibiotics (prescribed/not prescribed).
(f) Anticoagulants (prescribed/not prescribed).
(g) Steroids (prescribed/not prescribed).
(h) Mucolytics (prescribed/not prescribed).
(i) Bronchodilators (prescribed/not prescribed).
(j) Vitamins (prescribed/not prescribed).
(k) Antiplatelet agents (prescribed/not prescribed).

### 2.5. Data Sources

The data sources were patients’ medical histories taken from anonymous electronic clinical records (inpatient characteristics and diagnostic information at admission and treatment information from reports at patient discharge from a hospital) of the multidisciplinary city hospitals of the Al-Farabi Kazakh National University project conducted in Almaty, Astana, and Semey. The patient data collection period was 16 July 2022 to 31 January 2023.

### 2.6. Statistical Analysis

Numerical data are presented as means with standard deviation (sd). Absolute numbers with percentages summarize categorical variables. The normality of the data was tested using the Shapiro–Wilk or Kolmogorov–Smirnov test. The chi-squared test, Fisher’s exact test, independent-sample *t*-test, and Mann–Whitney U test were used to assess the differences in socio-demographic data, comorbidities, biochemical parameters, clinical treatment, and outcomes of the study population between pregnant and nonpregnant women.

To minimize potential biases in baseline characteristics between groups, a 1:1 propensity score matching (PSM) without replacement was applied. Propensity scores were calculated using a logistic regression model that included the following normalized covariates: severity of disease, residence status, and age category. In cases of missing covariate data, mean and frequency imputations were used. Patients in the nonpregnant group were matched to those in the pregnant group using nearest neighbor matching with a caliper width of 0.2 based on the pooled standard deviation of the logarithmic propensity scores. Standardized mean differences (SMDs) were computed to evaluate balance in baseline characteristics after matching, with an SMD of less than 0.1 considered an acceptable difference. The common support assumption was assessed using the Kolmogorov–Smirnov nonparametric test. Common support intervals were determined using the trimming method and kernel density estimators, applying a threshold of 0.001.

Univariate logistic regression analysis was used to assess predictors of death in the studied population. In all analyses, the significance level was set at 0.05. Statistical analysis was performed using IBM SPSS statistical software (SPSS for Windows, release 26.0, SPSS, Chicago, IL, USA).

## 3. Results

Table 1 presents a comparative analysis of the socio-demographic characteristics and clinical data of pregnant and nonpregnant women hospitalized with COVID-19. The data are reported for the raw (unmatched) sample, with each group consisting of 157 patients. Given that among infected pregnant patients were younger women, with severe COVID-19, and more from urban settings compared to nonpregnant women, we needed to match the sample to avoid potential biases in findings regarding the adverse COVID-19 outcomes. The matched sample included 78 pregnant and 78 nonpregnant women. After matching, key baseline differences, such as age and residence, were eliminated. Although more pregnant women were unemployed, this variable was not analyzed further because 70% of values were missing in the group of nonpregnant women.

After group matching, pregnant and nonpregnant women did not differ in their frequency of diarrhea, arrhythmia, respiratory rate, or heart rate compared to the total sample (Table 2). There was 80%–97% missing data on blood group in the database for pregnant and nonpregnant inpatients. After matching, anemia was more present in pregnant women than in nonpregnant women (73.1% vs. 19.2%), while significantly more prevalent among nonpregnant than pregnant inpatient were fever (97.4% vs. 69.2%), dyspnea (70.5% vs. 33.3%), myalgia (19.2% vs. 5.1%), chest discomfort (25.6% vs. 7.7%), sweating and weakness, higher median body temperature (38.3 °C vs. 37.5 °C), and fever above 38 °C (83.3% vs. 41.0%) (Table 2). Anosmia and diarrhea were reported exclusively in the nonpregnant group of inpatients with COVID-19 (nine and three cases, respectively).

Compared to nonpregnant patients, pregnant inpatients exhibited worse clinical characteristics both before and after the group matching, except for stage CT 2 and two-side CT process, which was worse for nonpregnant women before matching for severity, age, and residence to pregnant women (Table 3). After matching, a higher proportion of pregnant women presented with abnormal chest CT compared with nonpregnant women (6.0% vs. 2.9%), along with worse laboratory test results, including higher erythrocyte sedimentation rate, neutrophil proportion, platelet count, ALT, and AST. Additionally, pregnant women had elevated levels of leukocytes, lymphocytes, and C-reactive protein. D-dimer, creatinine, and procalcitonin levels were significantly higher in the pregnant group compared to the nonpregnant inpatients, with results showing 2402.97 ng/mL versus 793.91 ng/mL, 82.59 mmol/L versus 65.68 mmol/L, and 0.398 ng/mL versus 0.134 ng/mL, respectively (Table 3).

Based on the discharge reports, pregnant and nonpregnant patients were prescribed various medications, including antiviral treatments, antibiotics, corticosteroids, anticoagulants, mucolytics, bronchodilators, antiplatelet agents, and vitamins. These prescriptions adhered to the treatment guidelines for COVID-19 that were implemented during the study period in Kazakhstan. After group matching, significant differences were found only in prescribed antibiotics, corticosteroids, anticoagulants, and nasal cannulas between the matched groups of pregnant and nonpregnant women (Table 4). These disparities were understandable in light of the COVID-19 symptoms presented in Table 2. Specifically, pregnant women were prescribed antibiotics, anticoagulants, and steroids more frequently than their nonpregnant counterparts. In contrast, the prescription rate for nasal cannulas was significantly higher among nonpregnant women, 22.1%, compared to only 2.6% for pregnant women (Table 4).

After group matching, death cases were lost. Among other study outcomes of interest, a significantly higher proportion of pregnant women required ICU admission compared to nonpregnant women (16.9% vs. 2.6%). The differences in the ARDS and mechanical ventilation proportion between groups did not reach statistical significance (Table 5).

### Maternal Characteristics and Maternal, Pregnancy, and Neonatal Outcomes According to COVID-19 Severity (Moderate/Mild Versus Critical/Severe) Among Pregnant Inpatients

To address the second study objective, among all 157 pregnant inpatients, we analyzed maternal characteristics, as well as maternal, pregnancy, and neonatal outcomes, in relation to the COVID-19 severity. The mean gestational age of pregnant inpatients with moderate/mild COVID-19 was lower than that of pregnant women with critical/severe illness (26 ± 9.2 weeks versus 29.66 ± 6.4 weeks). Still, among pregnant women with critical/severe COVID-19, the overall rate of preterm birth was 40% and postpartum hemorrhage 10%. A higher proportion of pregnant women with critical/severe COVID-19 underwent cesarean delivery compared to those with moderate/mild COVID-19 (40% vs. 6.8%). The occurrence of stillbirth was 8.7% among cases with critical/severe and 5% in cases with moderate/mild COVID-19. All 13 deaths of pregnant women occurred in the group with critical/severe COVID-19, with the majority of deaths associated with critical COVID-19 (nine cases, 69.2%), and the mean gestational age at the time of death was 28.9 ± 4.8 weeks (Table 6).

Given that death cases were lost after group matching, univariate and multivariate logistic regression analyses were conducted on the complete sample (*n* = 314) to evaluate the clinical and laboratory factors significantly associated with mortality. Out of clinical pregnant and nonpregnant inpatient characteristics, significant factors independently associated with death incidence were as follows (Table 7): severity of disease, arterial hypertension, respiratory rate, heart rate, arrhythmia (*p* < 0.001 for all), and hypotonia (*p* = 0.002). Lower values of hemoglobin (*p* < 0.001) and higher values of erythrocyte sedimentation rate (*p* < 0.001), leukocytes (*p* = 0.005), segmented neutrophils (*p* < 0.001), band neutrophils (*p* = 0.004), AST (*p* < 0.001), procalcitonin (*p* < 0.001), CRP (*p* < 0.001), D dimer (*p* < 0.001), creatinine (*p* < 0.001), glucose (*p* < 0.001), ferritin (*p* < 0.001), CT stage (*p* < 0.001), and the use of bronchodilators (*p* = 0.009). However, according to the multivariate logistic regression analysis, none of those factors was a significant predictor of death (Table 7).

## 4. Discussion

The present study investigated the socio-demographic, clinical diagnostic, and treatment features associated with adverse COVID-19 outcomes among pregnant and nonpregnant Kazakhstani inpatients. In addition, these events were separately analyzed among pregnant in patients with mild/moderate and severe COVID-19. Previous studies showed that among pregnant women, a higher risk of developing COVID-19 infection was associated with socio-demographic variables such as older age [12,37], unemployment [27], severe COVID-19 [38,39]. Urban residence was positively associated with COVID-19 deaths among pregnant women in Nigeria [29]. To avoid potential biases, we applied propensity score matching for severity of COVID-19, age, and residence in the groups of pregnant and nonpregnant women. After group matching, the pregnant women diagnosed with COVID-19 were more likely to have common COVID-19 symptoms such as anemia and worse laboratory test results and CT diagnostics, which is consistent with previous studies analyzed in the systematic review and meta-analysis [14].

At admission to the hospital, fever and a temperature higher than 38 degrees Celsius were more frequently observed in the group of nonpregnant patients compared to pregnant inpatients in our study. This likely reflects pregnant women seldom ignoring even a slight increase in temperature. However, other authors found that a higher percentage of pregnant women had a temperature above 38 C than nonpregnant women [26]. We found several potential explanations from the available literature on conflicting information regarding fever prevalence in pregnant versus nonpregnant women with COVID-19: physiological changes during pregnancy may impact temperature regulation and immune responses. Pregnant women typically have a higher basal body temperature and altered thermoregulation, which can affect fever presentation [40]. Additionally, pregnancy-related immunological changes may modulate the inflammatory response to SARS-CoV-2 infection, potentially leading to differences in fever manifestation [41]. The timing of infection during pregnancy may play a role. Li et al. [40] found that patients infected with SARS-CoV-2 in early pregnancy presented similar laboratory tests to their nonpregnant peers, but as pregnancy progressed, increased inflammation markers became more frequent. This could explain variations in fever prevalence depending on the gestational age of the study participants.

Common laboratory changes observed in pregnant women included lymphopenia, leukocytosis, thrombocytopenia, and increased levels of C-reactive protein, D-dimer, ALT, AST, creatinine, and procalcitonin. Consistently with prior studies [42,43,44], in our study, laboratory characteristics showed significantly higher levels of inflammation markers such as white blood cell count, neutrophil percentage, C-reactive protein, procalcitonin, and D-dimer in pregnant women. Contrary to our findings, in other studies, the lymphocyte count was lower in pregnant women compared to nonpregnant ones [43,44,45,46]. The findings of our study, alongside those reported by Hazari et al. [42], demonstrated an increase in platelet counts among pregnant women diagnosed with COVID-19. The findings of another study reported an increase in platelet counts among nonpregnant women with COVID-19 [44]. The levels of liver enzymes in our results, including alanine transaminase (ALT) and aspartate aminotransferase (AST), were found to be significantly higher in pregnant patients with COVID-19 compared to nonpregnant patients with the disease. This elevation may reflect an increased susceptibility to liver dysfunction in pregnant women with COVID-19, highlighting the need for careful hepatic monitoring in this population. The present study demonstrates that both erythrocyte sedimentation rate (ESR) and creatinine levels were elevated beyond normal ranges in both groups analyzed. However, these levels were notably higher among pregnant women with COVID-19, suggesting a potentially heightened inflammatory response and renal stress in this population.

Pregnancy trimester and labor were important factors analyzed, because COVID-19 infection can affect pregnant women differently depending on the stage of pregnancy. Studies have shown that most cases are reported in the third trimester or during labor [47,48]. In our study, a careful examination of the group of pregnant inpatients with COVID-19 indicated that most critically severe pregnant women were in their third trimester, and the mode of delivery was a key variable for analysis. In our study, adverse outcomes from COVID-19, such as increased rates of cesarean sections, ARDS, premature birth, and postpartum hemorrhage, were more common in critically severe pregnant women. Some studies discovered that vaginal delivery, when compared to cesarean sections, was not linked to worse COVID-19 outcomes [49,50,51]. While some authors found higher rates of preterm delivery in COVID-19 patients [52], others found no significant difference [53]. This variability underscores the need for continued research on this outcome. Postpartum hemorrhage was analyzed to assess potential complications during and after delivery. While some studies found no significant differences in postpartum hemorrhage rates [53], others noted increased use of uterotonic agents in COVID-19 patients [54]. This variable’s frequency in analyses reflects the importance of monitoring for delivery-related complications in COVID-19 patients.

In our study, ICU admissions were more frequent among pregnant women than among nonpregnant women after group matching. In the UK, pregnant women accounted for 9% of all COVID-19 ICU admissions [55]. Equivalent adverse outcomes of COVID-19 in pregnant women observed by Deemah Salem and associates [56]: pregnant women were found to have a higher probability of hospitalization than nonpregnant women, particularly admission to the ICU for mechanical ventilation. However, the risk of mortality was similar in both groups. Similarly, Jafari and associates [14] found a significant need for ICU admission, with some cases requiring mechanical ventilation or ECMO. Many severe and critical pregnancies resulted in premature births, cesarean deliveries, and instances of postpartum bleeding. Additionally, mortality appeared to be higher in pregnant women compared to nonpregnant women [57,58].

Similarly, Allotey et al. [12] reported that 4% of pregnant women with COVID-19 were admitted to an ICU, 2% required invasive ventilation, and 0.2% required extracorporeal membrane oxygenation. They reported a significantly increased likelihood of all-cause mortality (odds ratio (OR) 6.09, 95% confidence interval (CI) 1.82–20.38) and ICU admission (OR 5.41, 95% CI 3.59–8.14) among pregnant and recently pregnant women with COVID-19. In a cohort study by Chinn and colleagues [59], pregnant women with COVID-19 experienced higher rates of adverse outcomes, including ICU admissions (5.2% vs. 0.9%), need for mechanical ventilation (1.5% vs. 0.1%), and mortality (0.1% vs. 0.01%). Sangam Jha and colleagues [60] identified a significantly increased risk of severe acute respiratory distress syndrome (ARDS) (33.3% vs. 23.4%), ICU admission (83.3% vs. 70%), and need for mechanical ventilation (66.6% vs. 48.9%) among pregnant women with COVID-19 compared to nonpregnant women.

Our study’s strengths and limitations have significant implications for policy and practice. The analyzed variables underscore the critical need for monitoring complications in COVID-19 patients. This research is founded on original data and aims to enhance the limited literature concerning pregnancy outcomes among COVID-19 patients within this region, informing clinical decision-making and ensuring safety in crises.

Additionally, this study aimed to elucidate the comparative impact of COVID-19 on pregnancy outcomes in Kazakhstan and analogous contexts. To facilitate international comparisons, we utilized universally accepted indicators to describe adverse outcomes. Furthermore, we concentrated on indicators pertinent to multidisciplinary hospital treatment technologies, which include diagnostic and treatment capabilities, resources, competencies, indications, structural developments, policies, and regional coverage, thereby minimizing potential biases.

It is important to emphasize that the findings are specific to the pandemic period and may have significant implications for case management in critical situations.

Due to the inherent characteristics of the study design and the methods employed for data collection, potential biases associated with the selected time frame and participating hospitals may have resulted in an overestimation or underestimation of the identified relationships. To mitigate this concern, we have provided 95% confidence intervals for our findings and propensity score matching of the compared groups.

While we analyzed a significant volume of data, certain confounding variables related to the study participants were outside the control of the researchers, which may affect the validity of our conclusions. For example, this study did not encompass outcomes for newborns, such as neonatal mortality and admission to the neonatal intensive care unit. We were also unable to analyze the vaccination status of pregnant women against COVID-19, as vaccination for pregnant women in Kazakhstan commenced on 15 November 2021, and relevant vaccination data were not available in electronic clinical records.

Furthermore, despite the multisite nature of the data collected, validation of our findings in a larger cohort in future research is necessary. Consequently, the generalizability of our conclusions may be limited to the specific population and settings examined in this study. To develop a more comprehensive understanding of the topic, it is essential to conduct larger-scale studies that involve diverse populations. Future research should aim to incorporate a wider array of variables and outcomes, as well as assess the impact of the studied factors on the health of newborns.

## 5. Conclusions

Pregnant women exhibited fewer common COVID-19 symptoms, such as fever, cough, and dyspnea, compared to nonpregnant women. However, research indicates that this population is at a heightened risk of adverse pregnancy outcomes. Pregnant women diagnosed with COVID-19 may exhibit more severe clinical symptoms and encounter less favorable outcomes compared to their nonpregnant counterparts. This was reflected in markedly elevated laboratory markers and CT stage severity. To effectively address these concerns, it is imperative to conduct early and regular monitoring of laboratory findings in pregnant individuals who are infected. This approach will enable healthcare providers to implement innovative interventions and evidence-based supportive measures more effectively, ultimately aiming to enhance health outcomes for both the mother and the fetus during this challenging period. Pregnant women were also more likely to require admission to the ICU. They faced a higher incidence of adverse outcomes, including acute respiratory distress syndrome, necessity for invasive and non-invasive mechanical ventilation, and mortality, though statistically insignificantly. Future studies should consider larger samples, individual variations in vaccination status, and the emotional and psychological impacts associated with their clinical condition.

## Figures and Tables

**Table 1:** Socio-demographic characteristics: pregnant and nonpregnant inpatients at admission to a hospital due to COVID-19 before and after propensity score matching.

Total Sample	Variable	Pregnant Women (*n* = 157)	Nonpregnant Women (*n* = 157)	*p*
Age (years)	mean ± sd	31.61 ± 5.6	37.88 ± 8.2	<0.001 ^a^
Age groups, *n* (%)	<40	151 (96.2%)	107 (68.2%)	<0.001 ^b^
	>40	6 (3.8%)	50 (31.8%)	
Severity of disease, *n* (%)	Mild and Moderate	117 (74.5%)	148 (94.3%)	<0.001 ^b^
	Severe and Critical	40 (25.5%)	9 (5.7%)	
Residence, *n* (%)	Urban	98 (63.2%)	51 (32.5%)	<0.001 ^b^
	Rural	57 (36.8%)	106 (67.5%)	
Groups matched with propensity score	Variable	Pregnant women (*n* = 78)	Nonpregnant women (*n* = 78)	*p*
Age (years)	mean ± sd	31.67 ± 5.95	32.85 ± 6.13	0.158 ^a^
Severity of disease, *n* (%)	Mild and Moderate	72 (92.3%)	72 (92.3%)	1.000 ^b^
	Severe and Critical	6 (7.7%)	6 (7.7%)	
Residence, *n* (%)	Urban	39 (50.0%)	39 (50.0%)	1.000 ^b^
	Rural	39 (50.0%)	39 (50.0%)	

^a^—Independent-sample *t* test; ^b^—chi-squared test.

**Table 2:** Clinical Diagnostics I: Pregnant and nonpregnant inpatients at admission to a hospital due to COVID-19, before and after propensity score matching.

Total Sample	Variable	Pregnant Women (*n* = 157)	Nonpregnant Women (*n* = 157)	*p*
Comorbidities, *n* (%)	Arterial hypertension	23 (14.7%)	16 (10.2%)	0.223 ^b^
	Diabetes	4 (2.8%)	3 (2%)	0.649 ^c^
	Hyperglycemia	18 (11.7%)	21 (13.4%)	0.653 ^b^
	Anemia	106 (67.9%)	31 (19.7%)	<0.001 ^b^
Common symptoms, at least once, *n* (%)	Fever	114 (72.6%)	152 (96.8%)	<0.001 ^b^
	Cough	137 (87.3%)	145 (92.4%)	0.136 ^b^
	Dyspnea	76 (48.4%)	118 (75.2%)	<0.001 ^b^
	Myalgia	7 (4.5%)	34 (21.7%)	<0.001 ^b^
	Anosmia	8 (5.1%)	19 (12.1%)	0.027 ^b^
	Diarrhea	0 (0%)	5 (3.2%)	0.024 ^c^
	Sweating	20 (12.7%)	30 (19.1%)	0.123 ^b^
	Weakness	133 (84.7%)	150 (95.5%)	0.001 ^b^
	Chest discomfort	18 (11.5%)	48 (30.6%)	<0.001 ^b^
	Sore throat	23 (14.7%)	14 (8.9%)	0.110 ^b^
	Hypotonia	12 (7.7%)	6 (3.8%)	0.141 ^b^
	Arrhythmia	14 (8.9%)	1 (0.6%)	0.001 ^c^
	Respiratory rate, bmp: median (IQR)	24 (20–24)	22 (20–23)	<0.001 ^d^
	Heart rate, bpm: median (IQR)	93 (83–107)	92 (80–98)	0.008 ^d^
	Temperature, degrees Celsius: median (IQR)	37.5 (36.9–38)	38 (38–38.5)	<0.001 ^d^
	Temperature >38 degrees Celsius (% of cases)	65 (41.4%)	131 (83.4%)	<0.001 ^b^
Groups matched with propensity score	Variable (yes)	Pregnant women (*n* = 78)	Nonpregnant women (*n* = 78)	*p*
Comorbidities, *n* (%)	Arterial hypertension	8 (10.4%)	9 (11.5%)	0.819 ^b^
	Diabetes	1 (1.4%)	1 (1.3%)	1.000 ^c^
	Hyperglycemia	3 (3.9%)	5 (6.4%)	0.719 ^c^
	Anemia	57 (73.1%)	15 (19.2%)	<0.001 ^b^
Common symptoms, at least once, *n* (%)	Fever	54 (69.2%)	76 (97.4%)	<0.001 ^c^
	Cough	70 (89.7%)	71 (91.0%)	0.786 ^b^
	Dyspnea	26 (33.3%)	55 (70.5%)	<0.001 ^b^
	Myalgia	4 (5.1%)	15 (19.2%)	0.013 ^b^
	Anosmia	0 (0.0%)	9 (11.54%)	0.003 ^c^
	Diarrhea	0 (0.0%)	3 (3.9%)	0.245 ^c^
	Sweating	10 (12.8%)	23 (29.5%)	0.011 ^b^
	Weakness	66 (84.6%)	76 (97.4%)	0.009 ^c^
	Chest discomfort	6 (7.7%)	20 (25.6%)	0.003 ^b^
	Sore throat	12 (15.4%)	11 (14.1%)	0.823 ^b^
	Hypotonia	4 (5.1%)	4 (5.1%)	1.000 ^c^
	Arrhythmia	4 (5.1%)	1 (1.3%)	0.367 ^c^
	Respiratory rate, bmp: mean ± sd	93.1 ± 16.74	90.23 ± 15.92	0.385 ^a^
	Heart rate, bpm: mean ± sd	22.1 ± 2.76	22.31 ± 4.18	0.194 ^a^
	Temperature, degrees Celsius: mean ± sd	37.50 ± 0.82	38.27 ± 0.71	<0.001 ^a^
	Temperature >38 degrees Celsius	32 (41.0%)	65 (83.3%)	<0.001 ^b^

^a^—Independent-sample *t* test, ^b^—chi-squared test; ^c^—Fisher’s exact test; ^d^—Mann–Whitney U test.

**Table 3:** Clinical Diagnostics II: Biochemical parameters of pregnant and nonpregnant inpatients at admission to a hospital due to COVID-19, before and after propensity score matching.

Total Sample	Pregnant Women (*n* = 157)	Nonpregnant Women (*n* = 157)	*p*
Hemoglobin, g/L, median (Q1–Q3)	97 (86–104)	125.3 (111–134.1)	<0.001 ^a^
Erythrocyte sedimentation rate, mm/h, median (Q1–Q3)	47 (37–55)	20 (15–30)	<0.001 ^a^
Leukocytes/L, median (Q1–Q3)	27.3 (18–28.3)	6.9 (5.5–8.54)	<0.001 ^a^
Segmented neutrophils, %, median (Q1–Q3)	81 (74–87.3)	63.22 (52.68–79.55)	<0.001 ^a^
Band neutrophils, %, median (Q1–Q3)	10 (6–11)	6 (6–6)	<0.001 ^a^
Lymphocytes, %, median (Q1–Q3)	41.1 (26–47.9)	33.9 (26.5–40.39)	<0.001 ^a^
Platelets (×10^3^/mL, median (Q1–Q3)	340 (299–390)	317.2 (187.5–472.4)	<0.001 ^a^
ALT U/L, median (Q1–Q3)	38.3 (17.4–59)	20.3 (14.8–26)	<0.001 ^a^
AST U/L, median (Q1–Q3)	49 (25.4–63)	24.2 (19.1–29.6)	<0.001 ^a^
Procalcitonin, ng/mL, median (Q1–Q3)	0.4 (0.3–0.48)	0.1 (0.1–0.1)	<0.001 ^a^
C-reactive protein, mg/L, median (Q1–Q3)	57.8 (25–113.7)	42.1 (12.3–66.4)	<0.001 ^a^
D-dimer, ng/mL, median (Q1–Q3)	1670 (1100–3960)	290 (120–675)	<0.001 ^a^
Creatinine, mmol/L, median (Q1–Q3)	89 (57–98.1)	63.4 (51.4–73)	<0.001 ^a^
Glucose, mmol/L, median (Q1–Q3)	6.1 (5.4–7.6)	6.14 (5.33–7.47)	0.833 ^a^
Ferritin, ng/mL, median (Q1–Q3)	69 (30–216)	100 (17.1–154.62)	0.963 ^a^
CT stage			
CT 1	65 (48.9%)	50 (35.2%)	0.005 ^b^
CT 2	47 (35.3%)	80 (56.3%)	
CT 3	13 (9.8%)	8 (5.6%)	
CT 4	8 (6%)	4 (2.8%)	
One side CT process	30 (20.5%)	15 (10.3%)	0.016 ^b^
Two-side CT process	116 (79.5%)	130 (89.7%)	
Characteristics in groups matched with propensity score	Pregnant women (n_p_ = 78)	Nonpregnant women (n_np_ = 78)	*p*
Hemoglobin, g/L, mean ± sd	97.55 ± 14.77	115.61 ± 20.47	<0.001 ^a^
Erythrocyte sedimentation rate, mm/h, mean ± sd	45.32 ± 12.01	22.17 ± 9.77	<0.001 ^a^
Leukocytes/L, mean ± sd	23.81 ± 5.78	7.45 ± 2.89	<0.001 ^a^
Segmented neutrophils, mean ± sd	75.69 ± 10.1	62.74 ± 16.78	<0.001 ^a^
Band neutrophils, %, mean ± sd	8.55 ± 5.44	5.92 ± 0.477	<0.001 ^a^
Lymphocytes, %, mean ± sd	38.55 ± 13.5	30.5 ± 13.17	<0.001 ^a^
Platelets, (×10^3^/mL), mean ± sd	337.1 ± 97.76	280.51 ± 168.37	<0.001 ^a^
ALT U/L, mean ± sd	42.88 ± 45.01	19.08 ± 6.77	<0.001 ^a^
AST U/L, mean ± sd	45.96 ± 44.67	28.97 ± 23.48	0.008 ^a^
Procalcitonin, ng/mL, mean ± sd	0.398 ± 0.264	0.134 ± 0.146	<0.001
C-reactive protein, mg/L, mean ± sd	63.74 ± 72.82	41.49 ± 53.22	0.005 ^a^
D-dimer, ng/mL, mean ± sd	2402.97 ± 2169.38	793.91 ± 1209.7	<0.001 ^a^
Creatinine, mmol/L, mean ± sd	82.59 ± 27.26	65.68 ± 24.33	<0.001 ^a^
Glucose, mmol/L, mean ± sd	6.07 ± 1.7	6.37 ± 2.67	0.541 ^a^
CT stage, *n* (%)			
CT 1	20 (29.9%)	39 (56.5%)	0.018 ^b^
CT 2	38 (56.7%)	24 (34.8%)	
CT 3	5 (7.5%)	4 (5.8%)	
CT 4	4 (6.0%)	2 (2.9%)	
One side CT process, *n* (%)	10 (14.3%)	8 (11.0%)	0.549 ^b^
Two-side CT process, *n* (%)	60 (85.7%)	65 (89.0%)	

^a^—Mann–Whitney U test, ^b^—chi-squared test.

**Table 4:** Differences in clinical treatment of pregnant and nonpregnant inpatients with COVID-19, before and after propensity score matching.

Treatment Total Sample	Pregnant Women (*n* = 157)	Nonpregnant Women (*n* = 157)	*p*
Transfusion of blood and blood substitutes	13 (8.3%)	2 (1.3%)	0.003 ^c^
Nasal cannula	12 (7.8%)	34 (21.8%)	0.001 ^b^
Extracorporeal membrane oxygenation	7 (4.5%)	1 (0.6%)	0.031 ^c^
Antibiotics	127 (80.9%)	104 (67.1%)	0.005 ^b^
Anticoagulants	145 (92.4%)	136 (87.2%)	0.131 ^b^
Steroids	103 (65.6%)	72 (46.2%)	<0.001 ^b^
Mucolytics	43 (27.4%)	45 (28.8%)	0.774 ^b^
Bronchodilators	12 (7.7%)	2 (1.3%)	0.006 ^c^
Vitamins	107 (68.6%)	98 (62.8%)	0.232 ^b^
Antiplatelets	19 (12.2%)	44 (28.2%)	<0.001 ^b^
Treatment in groups matched with propensity score	Pregnant women (*n* = 78)	Nonpregnant women (*n* = 78)	*p*
Transfusion of blood and blood substitutes	1 (1.3%)	1 (1.3%)	1.000 ^c^
Nasal cannula	2 (2.6%)	17 (22.1%)	<0.001 ^c^
Extracorporeal membrane oxygenation	1 (1.3%)	1 (1.3%)	1.000 ^c^
Antibiotics	57 (73.1%)	43 (56.6%)	0.032 ^b^
Anticoagulants	75 (96.2%)	60 (77.9%)	<0.001 ^c^
Steroids	46 (59.0%)	31 (40.3%)	0.020 ^b^
Mucolytics	13 (16.7%)	15 (19.5%)	0.649 ^b^
Bronchodilators	2 (2.6%)	1 (1.3%)	1.000 ^c^
Vitamins	49 (62.8%)	41 (53.3%)	0.227 ^b^
Antiplatelet agents	10 (12.8%)	19 (24.7%)	0.059 ^b^

^b^—chi-squared test; ^c^—Fisher’s exact test.

**Table 5:** Study outcomes of interest: pregnant versus nonpregnant inpatients with COVID-19, before and after propensity score matching.

Adverse COVID-19 Outcomes in Total Sample	Pregnant Women (*n* = 157)	Nonpregnant Women (*n* = 157)	*p*
ICU admission	45 (28.7%)	3 (1.9%)	<0.001 ^c^
ARDS	20 (12.7%)	4 (2.5%)	<0.001 ^c^
Mechanical ventilation (invasive, non-invasive)	21 (17.9%)	4 (2.6%)	<0.001 ^c^
Death	13 (8.3%)	2 (1.3%)	0.003 ^c^
Adverse COVID-19 outcomes in the groups matched with propensity score	Pregnant women (*n* = 78)	Nonpregnant women (*n* = 78)	*p*
ICU admission	13 (16.9%)	2 (2.6%)	0.005 ^c^
ARDS	5 (6.7%)	1 (1.3%)	0.112 ^c^
Mechanical ventilation (invasive, non-invasive)	5 (8.3%)	2 (2.7%)	0.242 ^c^

^c^—Fisher’s exact test.

**Table 6:** Maternal characteristics and maternal, pregnancy, and neonatal outcomes according to COVID-19 severity among pregnant inpatients (moderate/mild versus critical/severe).

Characteristics	Pregnant Women (*n* = 157)		*p*
	Moderate/Mild (*n* = 117)	Critical/Severe (*n* = 40)	
Gestational age on hospital admission due to COVID-19 (weeks, days)	26 ± 9.2	29.66 ± 6.4	0.023 °
First trimester (1–13 w, 6 d)	12 (10.3%)	1 (2.5%)	0.032 ^a^
Second trimester (13 w, 6 d–27 w, 6 d)	44 (37.6%)	9 (22.5%)	
Third trimester (28–42)	61 (52.1%)	30 (75%)	
Delivery events			<0.001 ^a^
Continued pregnancy	91 (82%)	14 (38.9%)	
Preeclampsia	1 (0.9%)	2 (5.7%)	0.134 ^c^
Premature birth	4 (3.4%)	16 (40%)	<0.001 ^a^
Mode of delivery			
Vaginal	6 (5.1%)	6 (15%)	0.001 ^a^
Cesarean	8 (6.8%)	16 (40%)	<0.001 ^a^
Postpartum events			
Hemorrhage	0 (0%)	4 (10%)	<0.001 ^a^
Stillbirth	1 (5%)	2 (8.7%)	0.554 ^c^
Neonates admitted to the neonatal department	0 (0%)	1 (4.2%)	0.545
Deaths among pregnant women	0 (0%)	13 (100%)	
Gestational age at death, w	-	28.9 ± 4.8	
Severe cases of COVID19 among deaths	-	4 (30.8%)	
Critical cases among deaths	-	9 (69.2%)	

^a^ Chi-squared test, ^c^ Fisher’s exact test, ° independent-sample *t*-test.

**Table 7:** Univariate and multivariate logistic regression analysis with death as dependent variable in the total sample of inpatient women with COVID-19 (*n* = 314).

Variable	Univariate Logistic Regression Analysis		Multivariate Logistic Regression Analysis	
	Odds Ratio	95% Confidence Interval	Odds Ratio	95% Confidence Interval
Severity of disease	58.57	13.10–261.79	>1000	0–>1000
Arterial hypertension (in anamnesis)	7.27	2.47–21.39	>1000	0–>1000
Respiratory rate, bmp	1.09	1.06–1.13	1.22	0–>1000
Heart rate, bmp	1.41	1.22–1.62	3.80	0–>1000
Arrhythmia (in anamnesis)	14.40	4.15–50.01	>1000	0–>1000
Hypotonia (in anamnesis)	7.38	2.08–26.11	0	0–>1000
Hemoglobin, g/L	0.95	0.92–0.97	1.63	0–>1000
Erythrocyte sedimentation rate, mm/h	1.08	1.04–1.12	0.72	0–>1000
Leukocytes/L	1.09	1.03–1.16	0.41	0–>1000
Segmented neutrophils, %	1.17	1.08–1.28	0.55	0–>1000
Band neutrophils, %	1.08	1.03–1.14	0.01	0–>1000
AST, U/L	1.02	1.01–1.02	0.93	0–>1000
Procalcitonin, ng/mL	4.33	2.00–9.41	1.14	0–>1000
C-reactive protein, mg/L	1.02	1.01–1.02	0.89	0–>1000
D-dimer, ng/mL	1.00	1.00–1.00	1.00	0–>1000
Creatinine, mmol/L	1.03	1.02–1.05	1.37	0–>1000
Glucose, mmol/L	1.34	1.16–1.54	0.01	0–>1000
Ferritin	1.00	1.00–1.01	1.04	0–>1000
CT stage (CT 1, 2, 3, 4)	4.66	2.51–8.66	90.28	0–>1000
Bronchodilators (prescribed)	6.52	1.61–26.48	0.00	0–>1000

## Data Availability

All data are presented in this paper. Original data are available from the corresponding author upon reasonable request.

## Abbreviations

The following abbreviations are used in this manuscript:

CT	computerized tomography
SpO2	percentage saturation of hemoglobin with oxygen
ARDS	acute respiratory distress syndrome
PCR	polymerase chain reaction
ICU	intensive care unit
BMI	body mass index
ALT	alanine aminotransferase
AST	aspartate aminotransferase
ECMO	extracorporeal membrane oxygenation
